# Oesophageal adenocarcinoma metastasis into a microcystic meningioma: a rare occurrence of tumour to tumour metastasis

**DOI:** 10.1259/bjrcr.20210157

**Published:** 2021-12-14

**Authors:** Basil Zia Khan, Oluwaseun Akinjise-ferdinand, Bhaskar Kumar

**Affiliations:** 1 Oxford University Hospital NHS Foundation Trust, Lancaster University, Lancaster, UK; 2 Royal Hallamshire Hospital, Sheffield Teaching Hospitals NHS Trust, Sheffield, UK; 3 Norfolk and Noriwch University Hospital, Norwich, UK

## Abstract

A 78-year-old male was admitted with a history of a fall following seizures. This occurred 2 years post-curative treatment (minimally invasive oesophagectomy with neo-adjuvant chemotherapy) for an oesophageal adenocarcinoma staged T3N0M0. On examination, patient had left-sided hemiparesis. A CT and magnetic resonance image (MRI) of the head confirmed a right frontotemporal meningioma with features suggestive of internal haemorrhage or calcification and mild local mass effect. A joint decision was made between the local neuro-surgical and neurology departments to manage this conservatively. However, due to progressive neurological deterioration and a concomitant increase in the size of the haemorrhagic lesion, emergent surgical intervention was indicated. The patient underwent a Simpson one complete resection (complete tumour resection including associated dura matter and abnormal underlying bone). Postoperative histology confirmed a rare case of metastatic oesophageal adenocarcinoma to a microcystic meningioma (World Health Organization Grade I). The meningioma was the only known site of distant metastasis for the oesophageal adenocarcinoma. Our case highlights the only documented case of the adenocarcinoma subtype of oesophageal tumour metastasizing to a meningioma. This case demonstrates the rare but well-documented occurrence of tumour to tumour metastasis. It highlights the importance played by imaging and clinical correlation when assessing progressively growing meningiomas in patients with a history of or underlying malignancy.

## Case presentation

**Figure 1.**
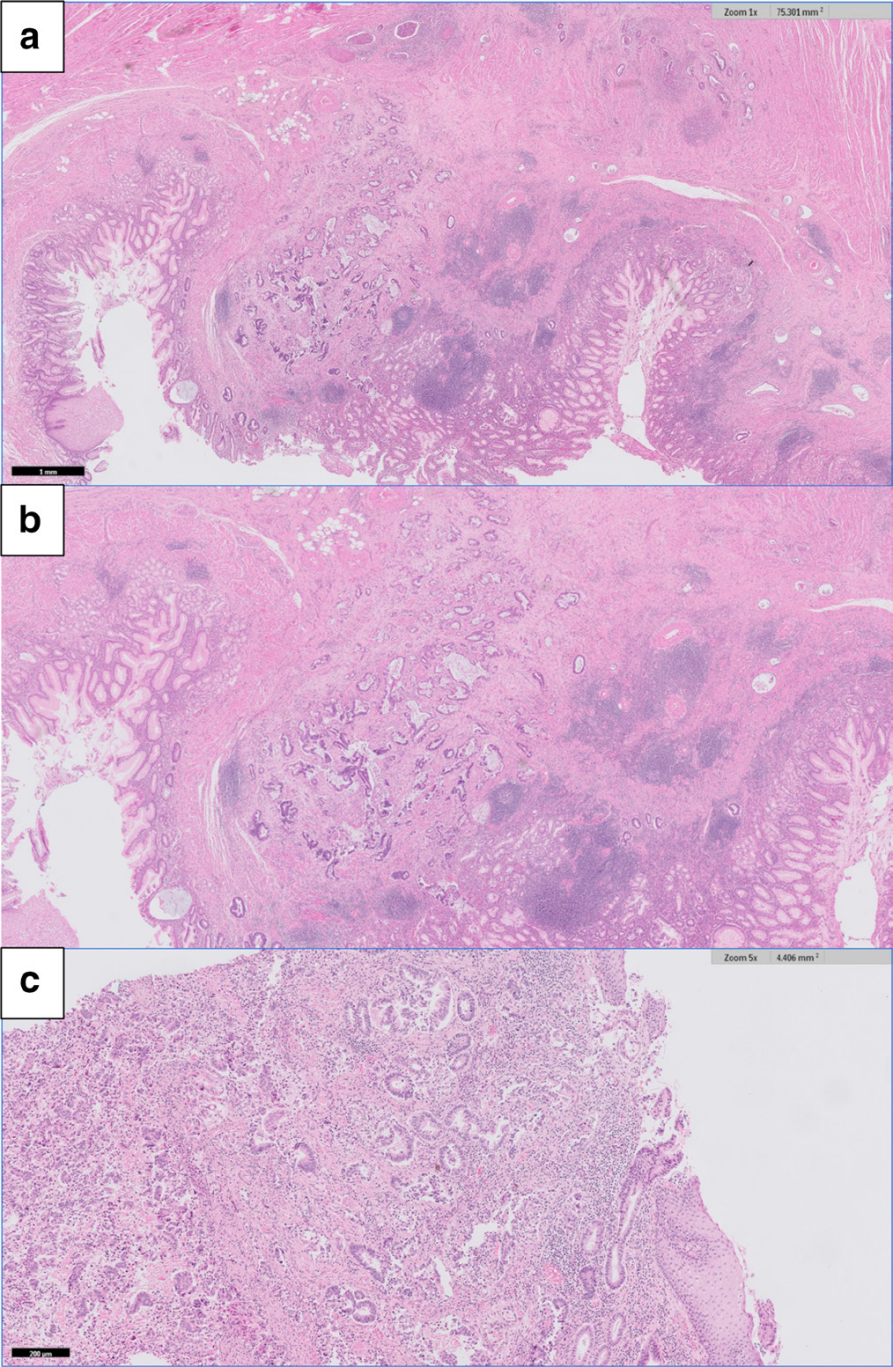
(A–C) Histology of Resected Oesophageal Adenocarcinoma showing extensive lymphovascular and perineural invasion of a moderately differentiated oesophageal adenocarcinoma

A 78-year-old gentleman was initially admitted on account of progressive dysphagia and weight loss. He had a past medical history of hypertension, hepatitis A, atrial fibrillation, Benign Prostatic Hyperplasia and a previous pulmonary embolism. A CT scan confirmed a lower third oesophageal carcinoma (T3N0M0). Following multidisciplinary team (MDT) confirmation of treatment of curative intent, he completed three cycles of Epirubicin, cisplatin and capecitabine (ECX) chemotherapy followed by a minimally invasive oesophagectomy. Figure 1 illustrates the histological appearance of the resected tissue. Patient made a good recovery from surgery and subsequently received three cycles of adjuvant ECX chemotherapy. Histology of excised mass revealed that the oesophagus contained a moderately differentiated (Grade 2) adenocarcinoma, Mandard regression grade was 3 (TRG 3).The patient attended for 3 monthly follow-up in the surgical oncology clinic and remained well.

Two years following oesophagectomy, the patient was admitted twice in a six-month period with a fall and repeated episodes of left-sided focal seizures and hemiparesis. On admission, examination revealed a Glasgow Coma Scale (GCS) of 15, antalgic gait, intact cranial nerves and a normal motor and sensory neurological exam of the upper and lower limbs. Imaging with CT and MRI on both occasions confirmed an atypical meningioma.

## Investigations

Computed Tomography (CT) Head scan on the first admission revealed a 34 × 32 × 23 mm, enhancing extra-axial lesion over the right posterior frontal region with associated vasogenic oedema. The appearances were highly suggestive of an atypical meningioma. MR Brain imaging showed an avidly enhancing 3.5 × 3.6 cm extra-axial mass overlying the right frontal temporal lobe. Mild mass effect on the adjacent cortex with some mild subcortical white matter oedema was seen.

**Figure 2.**
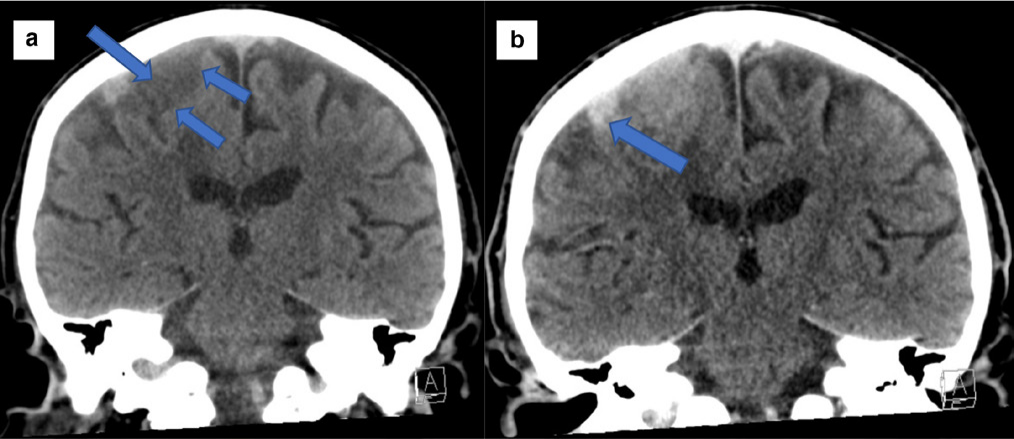
(A) Pre-Contrast Coronal CT Head demonstrating a 34 × 32 × 23 mm area of low attenuation soft-tissue extradural mass (Blue Arrow), which is overlying the superior right parietal lobe at the vertex. There is also a moderate degree of mass effect exerted upon the underlying cortex with sulcal effacement. (B) Post Contrast Coronal CT Head demonstrates relatively high-attenuation material (75 H.U.) within the lesion (Blue Arrow)

**Figure 3.**
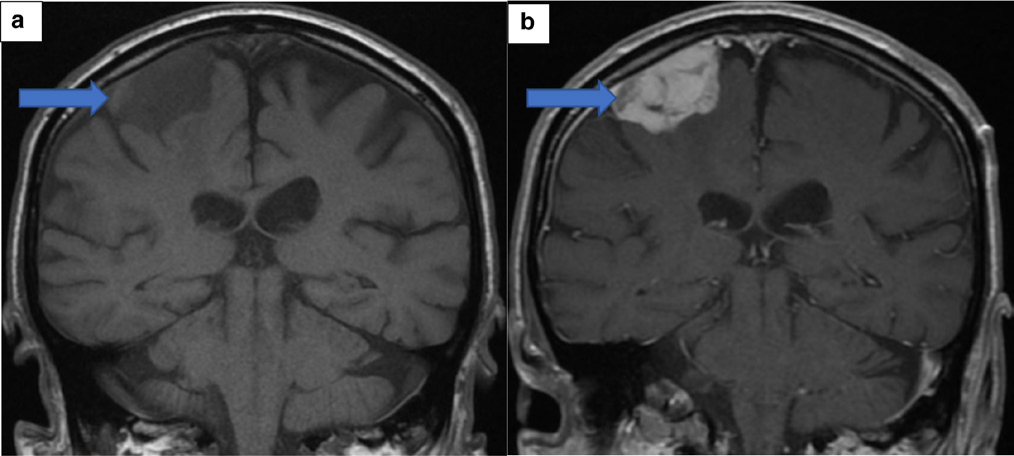
(A) Pre-Contrast Coronal T1 MRI Brain. (B) Post-Contrast Coronal T1 MRI. These images show a small focus of high T1 signal within at the right lateral aspect of the mass. This does not enhance, but demonstrates susceptibility artefact. This corresponded to the high density seen on CT. This represented the area of metastatic deposit within the meningioma

**Figure 4.**
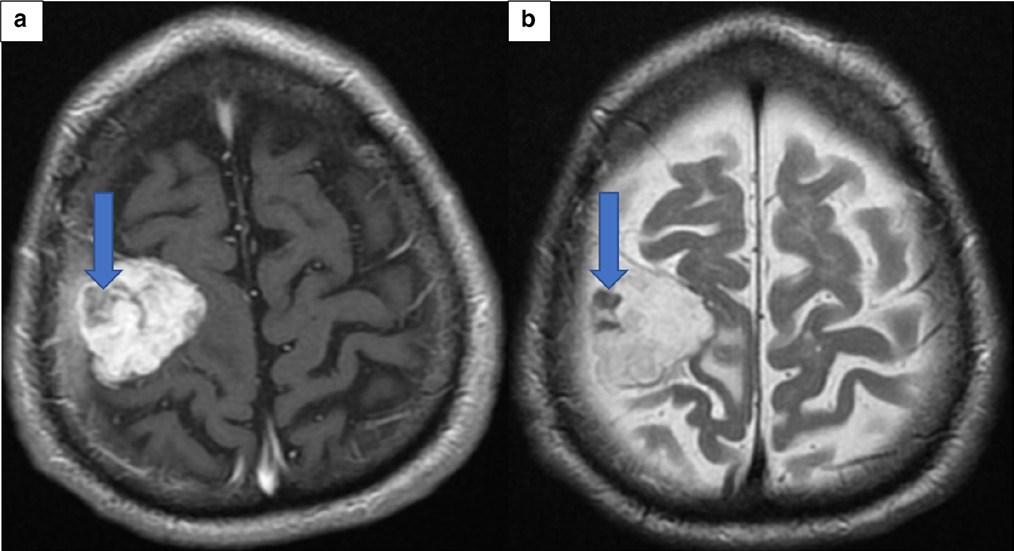
(A) Post-Contrast T1 Axial MRI Brain. (B) Post-Contrast T2 Axial MRI Brain. These demonstrate an area which does not enhance within the meningioma on both T1- and T2-weighted images (Blue Arrow). This area represented the metastatic deposit

On the second admission, a repeat CT head scan showed an interval increase in the volume of presumed haemorrhage within the known mass overlying the right parietal lobe (Figure 2). The mass itself remained unchanged in size, as was the degree of perilesional oedema and mass effect. A repeat MR brain further confirmed the CT head findings and was highly suggestive of an increasing haemorrhage within the right lateral aspect of the meningioma (Figures 3 and 4).

## Differential diagnosis

Based on the clinical presentation, examination and imaging the suspected diagnosis in our case was an atypical meningioma. Brain imaging was able to identify an area of a different density within the meningioma. The interval increase of volume of this area and progression of symptoms made a haemorrhage within the meningioma an important differential.

## Treatment

**Figure 5.**
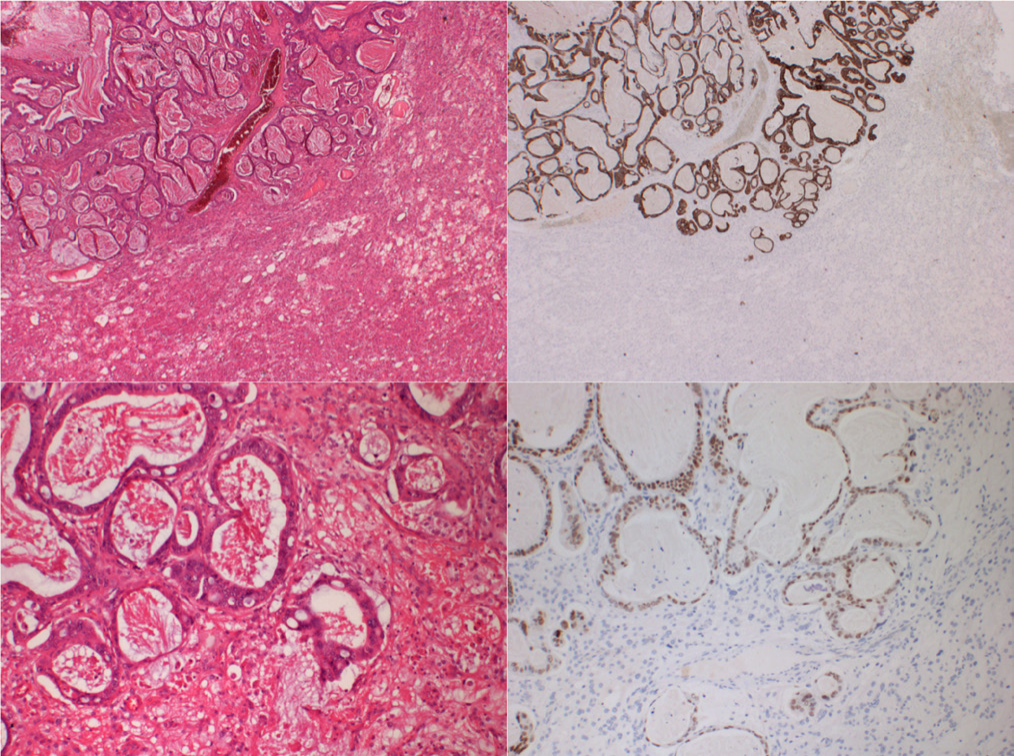
Histopathology of Excised Meningioma a. H&E (x20) showing collision between metastatic adenocarcinoma (top left) and microcystic meningioma b. Immunohistochemistry for cytokeratin 20 (x20) is strongly positive in the metastasis and negative in the meningioma. c. H&E (x100) showing border between metastatic adenocarcinoma and microcystic meningioma. d. Immunohistochemistry for CDX-2 (x100) showing nuclear positivity in the metastasis, consistent with a gastrointestinal origin

Initially, a decision was made to manage the meningioma conservatively with medical treatment and watchful waiting. Levetiracetam was commenced to control seizures. The patient continued to deteriorate despite medical management. This alongside repeat imaging resulted in a decision to excise the tumour. A Simpson one complete resection of the meningioma was done. No intra-operative images were taken however the tumour was reported to have an extra-axial firm appearance with no macroscopic features of distinct tumour components. Histology of the resected tumour revealed features suggestive of a microcystic meningioma with no evidence of brain invasion. Near its dural attachment, a smaller portion of the tumour consisted of a metastatic mucinous adenocarcinoma which was composed of cribriform and serrated acini of malignant columnar epithelium. These features were suggestive of a metastatic adenocarcinoma consistent with an oesophageal origin. The adenocarcinoma expressed CK7, CK2O and CDX-2 consistent with his previously known oesophageal primary. Figure 5 shows the histology of the resected tissue. There was no documentation of calcifications or haemorrhage within the excised tissue. The histological findings correlated well with the imaging done. The scans were able to identify the area of metastatic deposit within the meningioma which was confirmed by histology.

## Outcome/follow-up

Following surgical intervention, presenting symptoms resolved with no evidence of residual neurological deficit. Patient went on to make a full recovery and is fit and well at present. A recent CT Head, Thorax, Abdomen and Pelvis showed no recurrence of disease in the brain or oesophagus but noted multiple small slowly enlarging lung nodules which were too difficult to further characterize. In view of the patients medical history and CT findings, the oncology team have planned to observe and follow-up with a repeat CT scan at 3 months to assess for disease recurrence.

## Discussion

Tumour-to-tumour metastasis (TTM) is a rare but well-documented phenomenon. Literature to date has attempted to provide plausible explanations for the occurrence of this phenomenon; however, a clear understanding remains an area of active research and further study. One of the main challenges of TTM remains in its diagnosis. The relative rarity combined with the complexity of this condition has provided a challenge to researchers in providing a robust and widely accepted method of diagnosis.

One of the proposed hypothesis for the occurrence of TTM is the “seed and soil” theory. This proposes cross-talk between selected cancer cells (the “seeds”) and specific organ microenvironments (the ’soil')^1,2^ which leads to growth of metastasis within another tumour. Since the introduction of this theory over a century ago homeostatic factors, which promote tumour-cell growth, survival, angiogenesis and invasion have been identified to play a part in the occurrence of a TTM.^2^ Research has also suggested that high vascularity and locations of recipient sites play a role in the process of a metastasis situating itself within another tumour.^3^ This is known as the “mechanical theory”.

Meningiomas may provide the ideal “soil” given their low metabolic rate and abundance of lipids and collagen. Inflammatory infiltration is rare, allowing for the survival of metastatic cell colonies.^4–6^ Along with these features, meningiomas are slow-growing and relatively innocuous tumours at the onset, which allow for them to be ideal recipients for metastatic disease.^7^

Table 1 highlights three separate attempts to provide a method of establishing a diagnosis of TTM. Previous studies on TTM have used either of these proposed criteria to establish this rare diagnosis.^8^ Our proposed case fulfils all the criteria put forth by Campbell, Pamphlett and Dewan et al.

**Table 1.** Diagnostic Criteria for TTM^8–10^

	Criteria 1	Criteria 2	Criteria 3	Criteria 4
Campbell et al^8^	More than one primary co-exist	Recipient tumour must be true neoplasm	Donor neoplasm grows within recipient, not adjacent	Metastasis to lymphatic system where lymph reticular tumours pre-exist are excluded
Dewan et al^9^	Distant tumours mix with primary intracranial tumours	Histologic confirmation of two different tumours	Separate tumours presenting as one CNS lesion	
Pamphlett et al^10^	The foci of donor tumour should be surrounded by receipting tumour on histopathology	Confirmation of existing primary tumour by histology		

A definitive diagnosis of TTM with meningioma may be difficult via conventional CT or MRI imaging. A metastatic lesion within a meningioma may appear as a hyperdense area or, when associated with a necrotic component, as a hypodense area.^11^ Other features such as calcification, haemorrhage and cysts have been reported to be suggestive of TTM.^12^ However, it is important to note these findings are quite commonly seen in atypical meningiomas.

Microcystic meningiomas have been reported to have intense homogenous enhancement on CT, low intensity on *T1W* MR and a high signal with associated oedema on *T2W* MR scans.^13^ Our CT and MR scans demonstrated a difference in enhancement within the meningioma. This happened to correlate with the histopathological specimens obtained. Whereas the imaging was highly suggestive of a clear disparity within the meningioma, histological analysis was the only way of confirming this rare diagnosis. This point draws attention to the limit of CT and MR imaging when assessing tumours with a high suspicion of TTM.

FDG PET and MR Spectroscopy have been shown to demonstrate better analysis of meningiomas. FDG PET imaging allows for the identification of tissues with a high metabolic and glucose uptake rate. Meningiomas are typically indolent and slow-growing with a slow metabolic rate.^13^ A small area of increased uptake on an FDG PET scan within or next to a known meningioma could be suggestive of a TTM if there is a high suspicion. MR Spectroscopy has been used in one reported case to image a meningioma which was later confirmed to have an intra breast tumour metastasis.^14^ It has also been suggested that Perfusion MR may be more ideal in terms of identifying differing variants of a meningioma based on function, tissue metabolic composition and tumour vascularity respectively.^15,16^

Even although such imaging modalities may increase the diagnostic yield of TTM, a tissue diagnosis remains the gold standard diagnostic modality. The limit in imaging combined with the rarity of this pathology results in such changes in patients being put down to intra tumour haemorrhage or calcification rather than a TTM as these are more common. Despite this, the management at present remains the same regardless whether the presenting pathology is a simple meningioma or a more complex one such as a TTM (*i.e.,* observation, surgical excision ± radiotherapy). It is therefore important that when evaluating suspected TTM, pathologists should be conversant with the numerous meningioma variants, the possibility of an occult primary making its’ presence known, in addition to available medical history.^11^

In terms of treatment, radiotherapy and surgical resection are the most effective and widely used treatment modalities for meningiomas. The ability to achieve complete resection may be limited by a number of factors, including tumour location; involvement of nearby dural venous sinuses, arteries, cranial nerves, brain invasion into eloquent tissue and anaesthetics risks.^17^ Radiation therapy (RT) has been the primary treatment for nonsurgically resectable growing tumours. It is also used as adjuvant therapy (postresection) and in the setting of recurrence for previously resected meningiomas.

The histologic grade is the best predictive factor for local recurrence. The reported recurrence rates of Grades (I), (II), and (III) meningiomas are 7–25%, 29–52%, and 50–94%, respectively.^18^ The aggressive nature of grade II and III meningiomas may account for why the oesophageal squamous cell carcinoma metastasizing into an atypical meningioma described by Ritcher et al, carried a poorer prognosis in comparison to that described herein (Grade I).^17,19^ The National Institute of Clinical Excellence (NICE) has made recommendations on the follow-up of patients diagnosed with meningioma. It states an asymptomatic incidental meningioma should be followed up with a repeat scan at 12 months, if there is no change then to consider discharging the patient or repeating a scan at 5 years.^20^

In conclusion, tumour-to-tumour metastasis from a primary oesophageal carcinoma to meningioma’s are extremely rare. Challenges on diagnostics and management of this rare occurrence remain. Treatment and long-term outcomes of TTM have still not been determined and are an area of active research. Our case demonstrates the successful management of a patient presenting with adenocarcinoma of oesophageal origin metastasizing to a microcystic meningioma. It is the only documented case of the adenocarcinoma subtype of oesophageal tumour metastasizing to a meningioma.

## Learning points/take home message

- Oesophageal cancers metastasizing to a meningioma is an extremely rare occurrence but it does occur and can be managed effectively with good outcomes through an MDT approach.
- TTM should be considered as a differential diagnosis in patients with known or previous primary malignancy who are diagnosed with progressively growing meningioma or progressive neurological symptoms.
- More evidence is required in terms of identifying the role of MR Spectroscopy, FDG PET and Perfusion MR when evaluating an intracranial mass with high suspicion of TTM.
- Histology remains the mainstay of definitive diagnosis.
